# Analysis of the Transcriptional Differences between Indigenous and Invasive Whiteflies Reveals Possible Mechanisms of Whitefly Invasion

**DOI:** 10.1371/journal.pone.0062176

**Published:** 2013-05-08

**Authors:** Yong-Liang Wang, Yu-Jun Wang, Jun-Bo Luan, Gen-Hong Yan, Shu-Sheng Liu, Xiao-Wei Wang

**Affiliations:** Ministry of Agriculture Key Laboratory of Agricultural Entomology, Institute of Insect Sciences, Zhejiang University, Hangzhou, China; Kyushu Institute of Technology, Japan

## Abstract

**Background:**

The whitefly *Bemisa tabaci* is a species complex of more than 31 cryptic species which include some of the most destructive invasive pests of crops worldwide. Among them, Middle East-Asia Minor 1 (MEAM1) and Mediterranean have invaded many countries and displaced the native whitefly species. The successful invasion of the two species is largely due to their wide range of host plants, high resistance to insecticides and remarkable tolerance to environmental stresses. However, the molecular differences between invasive and indigenous whiteflies remain largely unknown.

**Methodology/Principal Findings:**

Here the global transcriptional difference between the two invasive whitefly species (MEAM1, MED) and one indigenous whitefly species (Asia II 3) were analyzed using the Illumina sequencing. Our analysis indicated that 2,422 genes between MEAM1 and MED; 3,073 genes between MEAM1 and Asia II 3; and 3,644 genes between MED and Asia II 3 were differentially expressed. Gene Ontology enrichment analysis revealed that the differently expressed genes between the invasive and indigenous whiteflies were significantly enriched in the term of ‘oxidoreductase activity’. Pathway enrichment analysis showed that carbohydrate, amino acid and glycerolipid metabolisms were more active in MEAM1 and MED than in Asia II 3, which may contribute to their differences in biological characteristics. Our analysis also illustrated that the majority of genes involved in ‘drug metabolic pathway’ were expressed at a higher level in MEAM1 and MED than in Asia II 3. Taken together, these results revealed that the genes related to basic metabolism and detoxification were expressed at an elevated level in the invasive whiteflies, which might be responsible for their higher resistance to insecticides and environmental stresses.

**Conclusions/Significance:**

The extensive comparison of MEAM1, MED and Asia II 3 gene expression may serve as an invaluable resource for revealing the molecular mechanisms underlying their biological differences and the whitefly invasion.

## Introduction

The whitefly *Bemisia tabaci* (Gennadius) (Aleyrodidae: Hemiptera) is a species complex consisting of at least 31 genetically diverse but morphologically indistinguishable cryptic species (hereafter species) [1]–[7]. Some members of this species complex are important pests of agricultural, horticultural and ornamental crops, causing extensive damage directly through phloem-feeding and indirectly through transmission of plant pathogenic viruses, primarily *begomoviruses* [8], [9]. Two species of the complex, Middle East-Asia Minor 1 (MEAM1) and Mediterranean (MED), which have been erroneously referred to as the B and Q ‘biotype’ respectively, have rapidly spread all over the world in the past 20 years [2], [10]–[13].

The MEAM1 species was presumed to originate in the Middle East-Asia Minor region and then quickly spread to more than fifty countries in six continents [2], [3], [11], [14]. Its invasive and damaging capacities have earned it a place as one of the world’s top 100 invasive species (http://www.issg.org). The MEAM1 species invaded China in the mid-1990s and were found in nearly all regions of China in 2011 [3], [15]–[17]. In Zhejiang province of eastern China, MEAM1 was first recorded in 2003 [18]. Subsequent field surveys indicated that the MEAM1 whitefly initially appeared in localities with frequent transport of plant materials and then gradually spread to the surrounding areas [13]. In many locations, the rapid spread of MEAM1 has occurred with the concurrent disappearance of the indigenous *B. tabaci* species [3], [13]. For example, before MEAM1 entered China, an indigenous whitefly Asia II 3 (previously known as ZHJ1) was the major species in many places of Zhejiang [13]. However, from 2004 to 2006, the population of Asia II 3 remarkably decreased and MEAM1 became the dominant species [13]. The same situation also occurred in Australia where the native whitefly species was replaced by MEAM1 [13]. Some efforts have been made to explore the possible factors responsible for the incursion of MEAM1 into new regions and the displacement of its closely related species [19]–[21]. Compared to indigenous species, MEAM1 has a wider range of host plants, higher resistance to insecticides and stronger tolerance to environmental stresses [2], [11], [21]–[24]. In addition, asymmetric mating interactions between MEAM1 and indigenous competitors have been shown to play a major role in the invasion of MEAM1 into China and Australia [13].

The MED species probably originated from the Mediterranean region and has been invading from its origin to other parts of the world [2], [25], [26]. It appeared in China in 2003 and its rapid spread in the following 6 years changed the composition of *B. tabaci* [3]. In some places, the MED species has successfully replaced both the native species and the invasive MEAM1, and become the only or predominant *B. tabaci* species in the field [3], [27], [28]. The competition between MED and MEAM1 is apparently mediated by many intrinsic and environmental factors [29]. In the last 10 years, MED’s higher level of resistance to neonicotinoid insecticides than MEAM1 has been shown to contribute to its displacement of the latter in China [29], [30]. In addition, MED’s higher level of resistance to pyriproxyfen and neonicotinoids than MEAM1 has also been suggested to be responsible for its relatively higher abundance in many regions in Israel and southern Spain [31], [32]. On the other hand, MEAM1 has a stronger capacity than MED for interspecific reproductive interference, which has been shown to contribute significantly to its displacement of MED in the laboratory [33], [34] and field [35]. These two species also differ in ranges of host plants, and such a difference seemed to have played a role in their competition and displacement as well [29]. However, due to the lack of genomic data, the molecular mechanisms underlying these biological and behavioral differences among the whitefly species remain largely unknown. Furthermore, previous studies have mainly focused on the behavioral and physiological differences of *B. tabaci* species. An overall description of the genetic factors associated with the competition and displacement between the invasive and indigenous whiteflies is still lacking.

In order to reveal the molecular mechanism of *B. tabaci* invasion, we compared the gene expression profiles of the invasive MEAM1, MED and the native Asia II 3 species using the Illumina sequencing technology. As gene expression difference is one of the major reasons leading to the divergence of species [36], studies on the variation of gene expression in different species can help unravel the molecular mechanisms that result in the biological and behavioral differences between species [37], [38]. In this study, we investigated the divergence of gene expression among the three whitefly species (MEAM1, MED and Asia II 3). The identification of differentially expressed genes among MEAM1, MED and Asia II 3 will serve as a valuable resource for future investigation of the molecular mechanisms underlying their biological variations and ability to invade.

## Materials and Methods

### Insect Culture

The invasive MEAM1 (mtCOI GeneBank accession no. GQ332577), MED (mtCOI GenBank accession no. DQ473394) and the indigenous Asia II 3 (mtCOI GenBank accession no. DQ309076) species of the *B. tabaci* species complex were collected from Zhejiang, China. No specific permits were required for the described field studies. The locations for sample collection are not privately-owned or protected in any way and the field studies did not involve endangered or protected species. Cultures of the three whitefly species were maintained separately on *Gossypium hirsutum* (Malvaceae) cv. Zhe-Mian 1793 in climate-controlled chambers at 27±1°C, a photoperiod of 14 h light: 10 h darkness and 70±10% relative humidity. The purity of the whitefly cultures was monitored every 3–5 generations using the random amplified polymorphic DNA polymerase chain reaction technique with the primer H16 (5′-TCTCAGCTGG-3′) than can differentiate *B. tabaci* genetic groups [39].

### Sample Preparation and RNA Isolation

To prepare pure whitefly cultures, a pair of virgin adult whiteflies of MEAM1, MED or Asia II 3 was separately released onto different cotton plants to oviposit. The eggs of each species were used to initiate a colony which was reared for five generations. To prepare whitefly samples, approximately 3,000 newly emerged whiteflies from the pure culture of each species were released onto the leaves of two cotton plants in different cages. After feeding for 24 h, approximately 500 female adults were separately collected for each species. Two biological replicates were collected for each species and processed independently. One replicate was used in the digital gene expression (DGE) [40] analysis and the other for the real-time quantitative PCR (qPCR) analysis. All experiments were conducted in climate-controlled chambers at 27±1°C, a photoperiod of 14 h light: 10 h darkness and 70±10% relative humidity. Total RNA was isolated using SV total RNA isolation system (Promega) according to the manufacturer’s protocol. RNA integrity was confirmed using the 2100 Bioanalyzer (Agilent Technologies) with a minimum RNA integrated number value of 8.

### DGE Library Preparation and Sequencing

The DGE method, which can generate absolute rather than relative gene expression measurements and avoid many of the inherent limitations of microarray analysis, was used to analyze gene expression variations among whiteflies of MEAM1, MED and Asia II 3 [41]. First, mRNA was purified from 6 µg of total RNA from each sample with magnetic oligobeads. First- and second- strand cDNA were synthesized and bead bound cDNA was subsequently digested with *NlaIII*, which recognizes the CATG sites. The cDNA fragments with 3′ ends were then purified with magnetic beads, and Illumina adapter 1 was added to their 5′ ends. The junction of Illumina adapter 1 and the CATG site is the recognition site of *MmeI*, which cuts 17 bp downstream of the CATG site, producing tags with adapter 1. After removing 3′ fragments with magnetic bead precipitation, Illumina adapter 2 was introduced at 3′ ends of tags, acquiring 21 bp tags with different adapters at both ends to form a tag library. Then, the three tag libraries of MEAM1, MED and Asia II 3 were sequenced in parallel using Illumina HiSeq 2000 platform at Beijing Genomics Institute (Shenzhen, China).

### Tag Annotation and Data Normalization for Gene Expression Level

**Figure 1:**
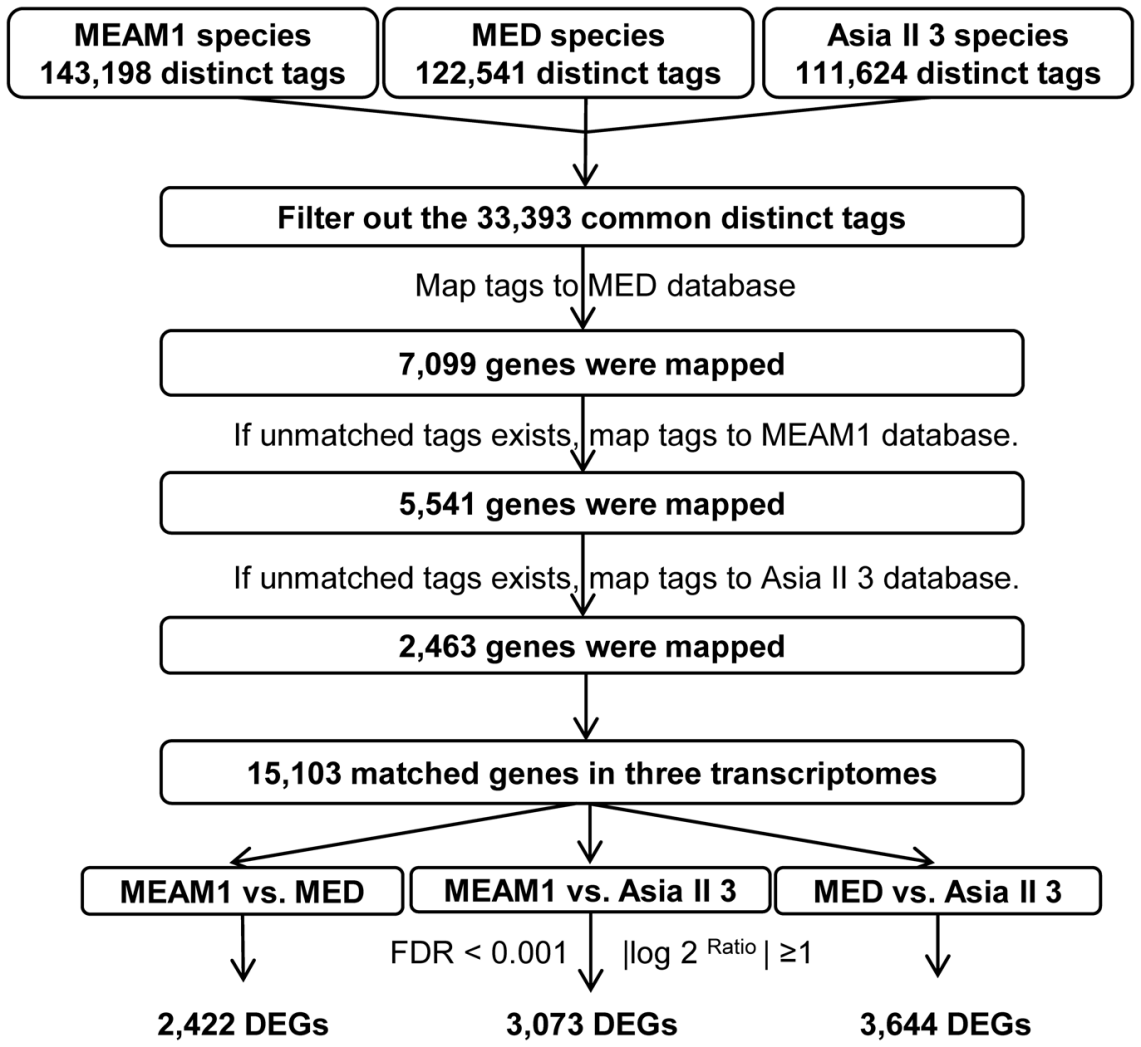
Approach used to identify DGEs among MEAM1, MED and Asia II 3 whiteflies. Common tags of MEAM1, MED and Asia II 3 species were first selected and mapped to the reference transcriptome database of MED. The unmatched tags were then mapped to the MEAM1 database. If unmatched tags still exist, we then mapped these tags to the Asia II 3 database. A total of 15,103 orthologous genes among MEAM1, MED and Asia II 3 were identified. Differentially expressed genes (DEGs) were identified between: i) MEAM1 and MED, ii) MEAM1 and Asia II 3, iii) MED and Asia II 3 with the threshold of FDR<0.001 and |log_2_ Ratio| ≥1.

Raw sequences were transformed into 21 bp clean tags through removing adaptor sequences, low quality sequences, empty reads and tags with a copy number of 1 (probably a sequencing error). Next, lists of all distinct 21 bp tags were generated from the MEAM1, MED and Asia II 3 sequencing results, respectively. As a distinct 21 bp tag corresponds to a unique gene in the transcriptome, the common tags of MEAM1, MED and Asia II 3 represent the orthologous genes among the three species. In order to identify the orthologous genes of MEAM1, MED and Asia II 3 which can be used for comparing the expression variation among the three species, the common tags of MEAM1, MED and Asia II 3 species were selected (Figure 1). Then, three reference databases of all possible CATG +17-nucleotide tag sequences in the transcriptome sequences of MEAM1, MED and Asia II 3 were created, respectively [42]–[44]. Next, the common tags of MEAM1, MED and Asia II 3 species were mapped to these three reference databases, respectively. Tags mapped to multiple reference sequences were filtered out, and the remaining tags were designated as unambiguous tags. Since the gene sequences among MEAM1, MED and Asia II 3 are highly conserved [42]–[44], the common tag mapped genes could be selected as the orthologous genes of MEAM1, MED and Asia II 3. To get the orthologous genes of MEAM1, MED and Asia II 3 from the MED transcriptomic database, all unambiguous common tags were mapped to the transcriptome reference database of MED and allowed no mismatch (Figure 1). As the MED transcriptome is incomplete, the unmatched common tags were then mapped to MEAM1 reference database to obtain more orthologous genes that were present in the MEAM1 transcriptome. If unmatched common tags still existed, we then mapped these tags to the Asia II 3 reference database to get the orthologous genes in Asia II 3 transcriptome (Figure 1). Finally, the orthologous genes from the three steps were put together to acquire the total orthologous genes of MEAM1, MED and Asia II 3 (Figure 1). Orthologous genes were annotated against NCBI nr database using an E-value cut-off of 1.0E^−5^. Functional annotation by Gene Ontology (GO) terms (http://www.geneontology.org) was analyzed by Blast2GO software. The nr blast results in xml format were imported to the software and GO annotation was performed on a local computer (about 3–4 hours). Pathway annotation was performed using Blastall software against the Kyoto Encyclopedia of Genes and Genomes (KEGG) database. For gene expression analysis, the numbers of unambiguous tags for each orthologous gene in the three libraries were calculated respectively and then normalized to the number of transcripts per million tags (TPM). DGE library data sets obtained in this work are available at the NCBI Gene Expression Omnibus under the accession number: GSE41864.

### Analysis of Differential Gene Expression

After knowing the orthologous genes and their expression levels in the three species, a rigorous algorithm was developed to identify differentially expressed genes (DEGs) between i) MEAM1 and MED, ii) MEAM1 and Asia II 3, iii) MED and Asia II 3 (Figure 1) [41]. False discovery rate (FDR) was used to determine the threshold of *p* value in multiple test and analysis. We used FDR<0.001 and the absolute value of log_2_ Ratio ≥1 as the threshold to judge the significance of gene expression difference (Figure 1). For an orthologous gene, the ratio means the normalized number of unambiguous tags contained in one species divided by the normalized number of unambiguous tags in another species. A total of three datasets were generated: 1) MEAM1 vs. MED that contains DEGs between MEAM1 and MED whiteflies; 2) MEAM1 vs. Asia II 3 that contains DEGs between MEAM1 and Asia II 3 whiteflies; and 3) MED vs. Asia II 3 that contains DEGs between MED and Asia II 3 whiteflies (Figure 1).

### GO and Pathway Enrichment Analyses

Pathway and GO enrichment analyses were performed to identify the significantly regulated pathways or GO terms between species. In pathway enrichment analysis, all genes were mapped to terms in the KEGG database and the pathways significantly enriched with DEGs were identified using hypergeometric test of R program. The R program has the function of ‘phyper’, so *p* = 1-phyper (m, n, N-n, M, lower. tail = TRUE, log. p = FALSE). The formula was defined as follows:
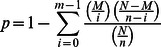
where N and n indicate the total numbers of genes and DEGs with KEGG annotations respectively, and M and m are the numbers of genes and DEGs annotated to a certain KEGG term. Pathways with *p*≤0.05 were deemed to be enriched with DEGs. In GO enrichment analysis, the hypergeometric test was used in a similar way to identify the GO terms enriched with DEGs (*p*<0.05). To increase the reliability, pathways or GO terms with less than five genes were filtered out.

### Real-time Quantitative PCR (qPCR) Analysis

To confirm the results of the DGE analysis, the expression of 30 selected genes (10 genes from each of the following three datasets MEAM1 vs. MED; MEAM1 vs. Asia II 3 and MED vs. Asia II 3) were measured using qPCR. Adult whiteflies of MEAM1, MED and Asia II 3 were collected according to the previous descriptions and total RNA was extracted. cDNA was synthesized using the SYBR®PrimeScript™ RT-PCR Kit II (Takara) and qPCRs were conducted on the ABI PRISM 7500 Fast Real-Time PCR System (Applied Biosystems) with SYBR-Green detection. Each gene was analyzed in triplicate and the average threshold cycle (C_T_) was calculated for each sample [45]. As an endogenous control, the expression of β-actin was measured in parallel. The expression levels of target genes were calculated with the comparative C_T_ method (also known as the 2^−ΔΔCT^ method), in which the gene expression levels is presented relative to the β-actin [46].

## Results and Discussion

### Summary of DGE Sequencing and Mapping

Three whitefly DGE libraries (MEAM1, MED and Asia II 3) were sequenced and approximately 6.0 million raw tags were obtained for each library (Table 1). After filtering out the low quality reads, the total number of tags per library ranged from 5.7 to 5.9 million and the number of distinct tags ranged from 111,624 to 143,198, which are available at the NCBI Gene Expression Omnibus under the accession number: GSE41864. Next, frequencies of distinct tags were used to evaluate the normality of DGE data [47]. Among the three samples, similar distributions of tag numbers were found at different categories of tag expression levels, suggesting that little bias exists in the construction of libraries from these three whitefly species (Figure S1). To identify the orthologous genes among the three species, which can be used for comparing gene expression, the 33,393 common distinct tags of MEAM1, MED and Asia II 3 sequencing libraries were filtered out and mapped to the MED, MEAM1 and Asia II 3 transcriptomes sequentially according to the procedures in Figure 1 (see Materials and Methods for details). After mapping, a total of 15,103 genes that represent the orthologous genes among the three species were identified. It is possible that different analytical approach might recover different numbers of orthologous genes.

**Table 1:** Statistics of digital gene expression sequencing.

Category		Parameter		MEAM1		MED	Asia II 3
Raw tag	Total tag number		595,1527		606,4401		611,4520
	Distinct tag number		342,727		290,999		247,567
Clean Tag	Total tag number		5,734,967		5,876,814		5,976,544
	Distinct tag number		143,198		122,541		111,624
Common tag	Total tag number		4,954,769		4,772,332		4,542,540
	Distinct tag number		3,3393		3,3393		3,3393

### Expression Level of the Orthologous Genes among the Three *B. tabaci* Species

**Figure 2:**
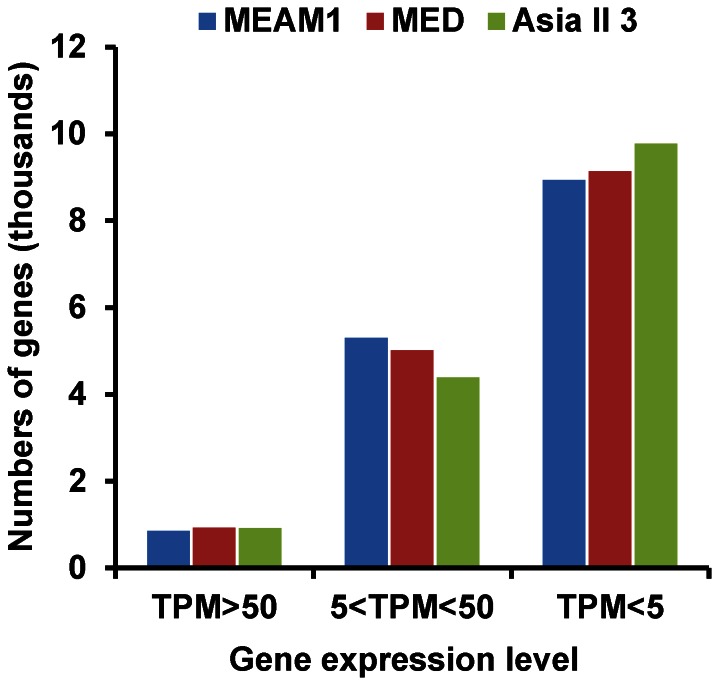
The distribution of gene expression level in MEAM1, MED and Asia II 3. The expression of genes was normalized to the number of transcripts per million tags (TPM).

For each species, the number of tags mapped to each gene was calculated and the expression level of these 15,103 orthologous genes in each species were determined respectively (Table S1). Next, genes were grouped into different categories based on the number of transcripts per million tags (TPM) and the distribution of genes in each category was analyzed (Figure 2). The results showed that, for each species, about 60% of the genes (59.2% in MEAM1, 60.6% in MED and 64.8% in Asia II 3) were expressed at a low level with TPM<5; whereas the genes with high expression level (TPM>50) only constituted a small portion (5.7% in MEAM1, 6.2% in MED and 6.1% in Asia II 3) (Figure 2). Then, the functions of the top 10 highly expressed genes with annotations were analyzed for each species. Many of the genes with very high expression level were involved in ribosome (e.g. 60S ribosomal protein) and energy metabolism (e.g. NADH dehydrogenase and cytochrome c oxidase subunit) (Table S1). This finding is not surprising, as these genes are essential for the survival of an organism. Interestingly, a CuZn superoxide dismutase (CuZnSOD) gene, which has been proved to be an important antioxidant in many insects [48], [49], was identified in the top 10 highly expressed genes of MED species. Interestingly, the expression level of CuZnSOD in MED (TPM = 578) was much higher than that in MEAM1 (TPM = 315) and Asia II 3 (TPM = 116) indicating that this gene may play an important role in MED (Table S1).

### Overview of the Differentially Expressed Genes (DEGs) Among the Three Species

**Figure 3:**
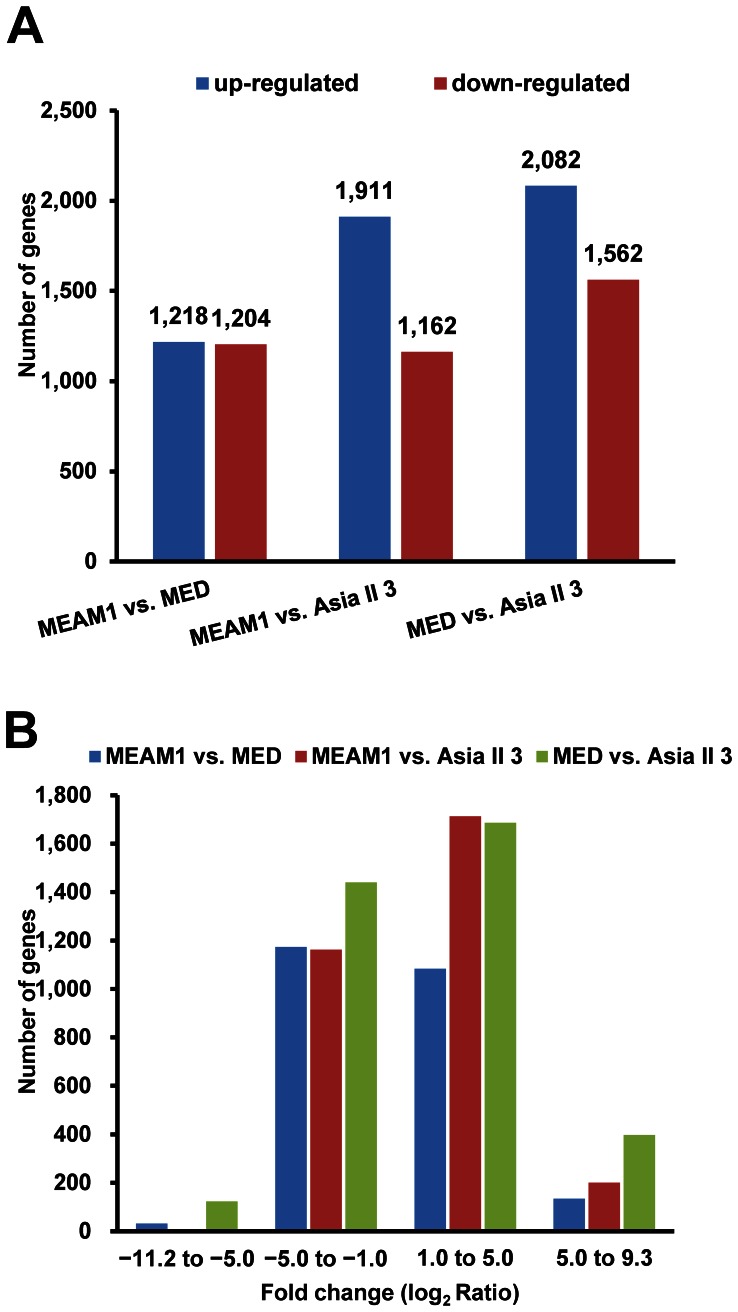
Differentially expressed genes among MEAM1, MED and Asia II 3. (A) The number of up-regulated and down-regulated genes among MEAM1, MED and Asia II 3. (B) Fold change (log_2_ Ratio) distribution of differentially expressed genes.

Because only the expression level of orthologous genes can be compared between different species, to identify the DEGs among MED, MEAM1 and Asia II 3, three comparisons were performed for the 15,103 orthologous genes: 1) MEAM1 vs. MED; 2) MEAM1 vs. Asia II 3; and 3) MED vs. Asia II 3. In this way, differences among the three species can be identified and compared based on the same dataset. This analysis identified 2,422 DEGs in MEAM1 vs. MED, 3,073 DEGs in MEAM1 vs. Asia II 3 and 3,644 DEGs in MED vs. Asia II 3 (Figure 1 and Table S2 in the supplemental material), which represent 16.0%, 20.3% and 24.1% of the 15,103 orthologous genes, respectively. The minimum number of DEGs between MEAM1 and MED (2,422) and the maximum number of DEGs between MED and Asia II 3 (3,644) were apparently coordinated with the phylogenetic analysis that MED have a relative closer relationship with MEAM1 than with Asia II 3 [1], [2]. Among the DEGs, 1,218 genes were up-regulated and 1,204 genes down-regulated in MEAM1 vs. MED; 1,911 genes were up-regulated and 1,162 genes down-regulated in MEAM1 vs. Asia II 3; and 2,082 genes were induced and 1,562 genes were repressed in MED vs. Asia II 3 (Figure 3A). In the three comparisons, the detected fold changes (log_2_ Ratio) of gene expression ranged from −11.2 to 9.3 folds (Figure 3B). The majority of DEGs were up- or down-regulated between 1.0- and 5.0- folds, whereas only a small portion of genes were up- or down-regulated more than 5.0- folds (Figure 3B). Interestingly, the number of genes with more than 5.0- folds differences in gene expression was much larger in MED vs. Asia II 3 (396 up- and 122 down-regulated genes) than in MEAM1 vs. MED (134 up- and 31 down-regulated genes) (Figure 3B). This result is also consistent with the phylogenetic analysis that MED is more closely related to MEAM1 than to Asia II 3.

To validate the DGE data, the gene expression was examined using qPCR. From each of these three comparisons (MEAM1 vs. MED, MEAM1 vs. Asia II 3 and MED vs. Asia II 3), 10 DEGs were selected for qPCR analysis. Out of the 30 genes selected, 27 demonstrated a concordant direction of change for both DGE and qPCR (see Table S3 and S4 in the supplemental material). The expression levels of the other 3 genes were inconsistent between DGE and qPCR results. The partial inconsistency may be caused by two reasons. First, due to the lower sensitivity of qPCR, minor differences in gene expression were difficult to be detected with qPCR assays. Second, the RNA samples used for qPCR and DGE were from different batches and the discrepancy might derive from different sample preparations. Nonetheless, the qPCR analysis confirmed that the DGE results were reliable. This result is consistent with a number of previously experiments, which have proved the validation of DGE analysis [45], [47], [50].

### Gene Ontology (GO) Analysis

**Figure 4:**
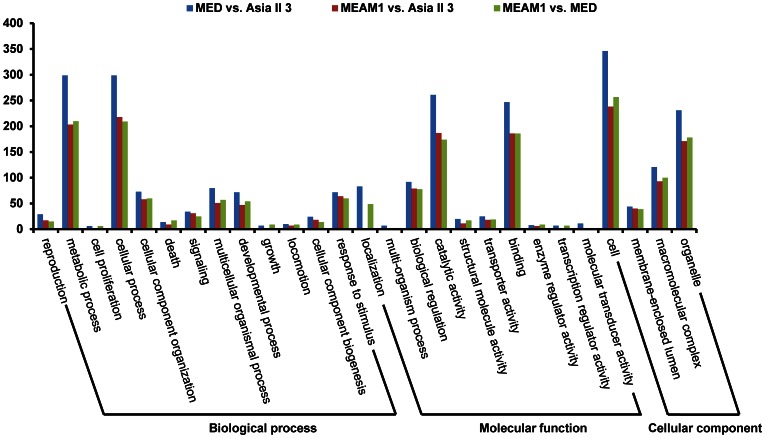
Histogram presentations of GO classification of DEGs among MEAM1, MED and Asia II 3. The functions of genes identified cover three main categories: Biological process, Cellular component, and Molecular function.

In order to reveal the functions of the DEGs in each of the three comparisons (MEAM1 vs. MED, MEAM1 vs. Asia II 3 and MED vs. Asia II 3), GO terms were assigned to all DEGs. In MEAM1 vs. MED, 278 genes were identified to the category of Biological process, 305 genes to Molecular function, and 261 genes to Cellular component. As for MEAM1 vs. Asia II 3, 278 genes were identified to Biological process, 302 genes to Molecular function, and 244 genes to Cellular component. For MED vs. Asia II 3, 394 genes were identified to Biological process, 412 genes to Molecular function, and 353 genes to Cellular component. Figure 4 showed that the overall GO annotations of DEGs in the three comparisons were very similar. The numbers of DEGs in each GO term are comparable among the three comparisons. Among the DEGs, the most represented GO terms in the category of Biological process are ‘cellular process’ and ‘metabolic process’, whereas ‘binding’ and ‘catalytic activity’ are the two most represented GO terms in the category of Molecular function.

### GO Enrichment Analysis

**Figure 5:**
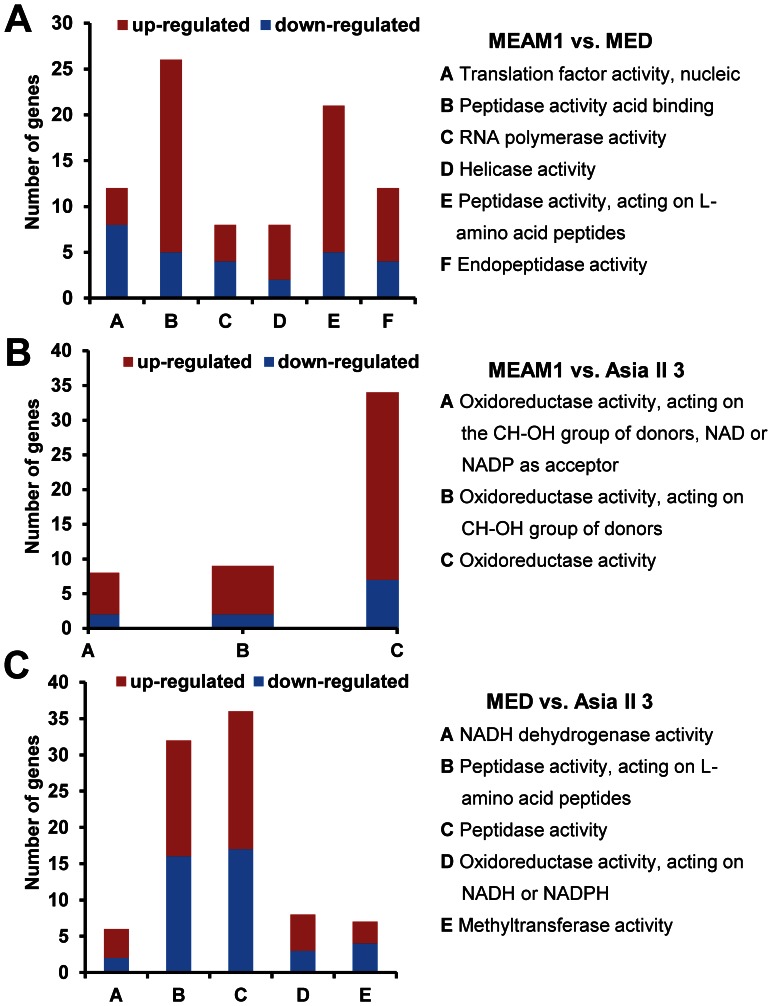
Analysis of DEGs in enriched GO terms. (A) The number of up-regulated and down-regulated genes in enriched GO terms between MEAM1 and MED. (B) The number of up-regulated and down-regulated genes in enriched GO terms between MEAM1 and Asia II 3. (C) The number of up-regulated and down-regulated genes in enriched GO terms between MED and Asia II 3.

To gain further insights into the function of DEGs, multilevel GO enrichment analysis was performed. Between MEAM1 and MED, DEGs were significantly enriched in 6 GO terms in the category of Molecular function (*p*<0.05) (Table 2). These GO terms were about ‘translation factor activity, nucleic acid binding’, ‘peptidase activity’, ‘RNA polymerase activity’, ‘helicase activity’, ‘peptidase activity, acting on L-amino acid peptides’, ‘endopeptidase activity’. Interestingly, three of the enriched GO terms were related to peptidase activity (Table 2) and the majority of DEGs in these GO terms were up-regulated in MEAM1 compared to MED (Figure 5A). It suggests that the peptidase activity might be higher in MEAM1 than in MED. As for MEAM1 vs. Asia II 3, all the enriched GO terms were about oxidoreductase activity and most of genes in those GO terms were up-regulated in MEAM1 compared to Asia II 3 (Table 2, Figure 5B). Regarding MED vs. Asia II 3, a total of 5 GO terms in the category of Molecular function were enriched, such as ‘oxidoreductase activity’, ‘peptidase activity’ and ‘NADH dehydrogenase activity’ (Table 2, Figure 5C). The data above revealed that the expression of genes in ‘peptidase activity’, ‘oxidoreductase activity’ and ‘NADH dehydrogenase activity’ were significantly different among the three species. Most importantly, in both MEAM1 vs. Asia II 3 and MED vs. Asia II 3, the expression of genes in ‘oxidoreductase activity’ were differentially regulated. Oxidoreductases are important factors that engage in various biochemical reactions such as energy generating, immunity and anti-oxidative stresses [51], [52]. The over-expression of genes in ‘oxidoreductase activity’ might be important for the high adaptability and invasion of MEAM1 and MED.

**Table 2:** Statistically enriched Gene Ontology terms among the DEGs in the ‘Molecular function’ category.

GO ID	Level	GO Ontology	No. of DEGs*	No. of genes**	*p*-value***
**MEAM1 vs. MED**					
GO:0008135	4	Translation factor activity, nucleic acid binding	12	45	0.025
GO:0008233	4	Peptidase activity	26	115	0.029
GO:0034062	6	RNA polymerase activity	8	29	0.037
GO:0004386	8	Helicase activity	7	25	0.041
GO:0070011	5	Peptidase activity, acting on L-aminoacid peptides	21	93	0.043
GO:0004175	6	Endopeptidase activity	12	49	0.048
**MEAM1 vs. Asia II 3**					
GO:0016491	3	Oxidoreductase activity	34	154	0.008
GO:0016614	4	Oxidoreductase activity, acting onCH-OH group of donors	9	28	0.010
GO:0016616	5	Oxidoreductase activity, acting on the CH-OHgroup of donors, NAD or NADP as acceptor	8	23	0.023
**MED vs. Asia II 3**					
GO:0003954	5	NADH dehydrogenase activity	6	9	0.001
GO:0070011	5	Peptidase activity, acting on L-aminoacid peptides	32	93	0.003
GO:0008233	4	Peptidase activity	36	115	0.009
GO:0016651	4	Oxidoreductase activity, acting on NADHor NADPH	8	19	0.014
GO:0008168	5	Methyltransferase activity	7	18	0.032

* The number of differentially expressed genes (DEGs) in a Gene Ontology (GO) term.

** The total number of orthologous genes in a GO term.

*** The significantly enriched GO terms in DEGs were identified using hypergeometric test and pathways with the p≤0.05 were deemed to be enriched in DEGs.

Next, enriched GO terms in the category of Biological process were also investigated (Table S5). The DEGs in MEAM1 vs. MED were enriched in 4 GO terms and the DEGs between MEAM1 and Asia II 3 were enriched in 6 GO terms. Surprisingly, the number of GO terms (26) enriched with DEGs between MED and Asia II 3 was more than 2 times of the total number of enriched GO terms in MEAM1 vs. MED (4) and MEAM1 vs. Asia II 3 (6). This result implied that in the category of Biological process the discrepancies between MED vs. Asia II 3 were much larger than MEAM1 vs. MED or MEAM1 vs. Asia II 3.

### Pathway Enrichment Analysis

To investigate which biological pathways were significantly regulated among the three species, we mapped their DEGs to KEGG pathways. Among the 2,422 DEGs in MEAM1 vs. MED library, 326 genes were mapped to 183 pathways. In MEAM1 vs. Asia II 3 library, 319 of the 3,073 DEGs were mapped to 189 pathways, and in MED vs. Asia II 3 library, 427 of the 3,644 DEGs were mapped to 205 pathways (Table S6). Enrichment analysis was subsequently conducted to identify the significantly influenced pathways (*p*<0.05). Between MEAM1 and MED, ten pathways were enriched and most of them were associated with carbohydrate and amino acid metabolisms such as ‘Galactose metabolism’, ‘Tyrosine metabolism’ and ‘Fructose and mannose metabolism’ etc. (Table 3). As for MEAM1 vs. Asia II 3, 11 pathways were enriched with DEGs (Table 4) and 15 pathways were enriched in MED vs. Asia II 3 (Table 5).

**Table 3:** Statistically enriched pathways among the DEGs between MEAM1 and MED.

Pathways	No. of DEGs*	No. of genes**	*p*-value***
Lysosome	28	83	0.000
Galactose metabolism	12	29	0.001
Tyrosine metabolism	6	16	0.014
Fructose and mannose metabolism	7	20	0.016
Polycyclic aromatic hydrocarbon degradation	6	18	0.028
Glutathione metabolism	9	30	0.029
Pentose and glucuronate interconversions	8	26	0.030
Pyruvate metabolism	8	26	0.030
Glycine, serine and threonine metabolism	5	15	0.036
Glycerolipid metabolism	7	24	0.049

* The number of differentially expressed genes (DEGs) that belong to a KEGG pathway.

** The total number of orthologous genes that belong to a KEGG pathway.

*** The significantly enriched pathways in DEGs were identified using hypergeometric test and pathways with the p≤0.05 were deemed to be enriched in DEGs.

**Table 4:** Statistically enriched pathways among the DEGs between MEAM1 and Asia II 3.

Pathways	No. of DEGs*	No. of genes**	*p*-value***
Histidine metabolism	5	11	0.006
beta-Alanine metabolism	6	15	0.008
Pentose and glucuronate interconversions	9	26	0.009
Tyrosine metabolism	6	16	0.013
Ribosome biogenesis in eukaryotes	16	59	0.020
Propanoate metabolism	6	18	0.026
Polycyclic aromatic hydrocarbon degradation	6	18	0.026
Ascorbate and aldarate metabolism	6	18	0.026
Drug metabolism - other enzymes	11	40	0.035
Glycerolipid metabolism	7	24	0.045
Methane metabolism	6	20	0.046

* The number of differentially expressed genes (DEGs) that belong to a KEGG pathway.

** The total number of orthologous genes that belong to a KEGG pathway.

*** The significantly enriched pathways were identified using hypergeometric test and pathways with the p≤0.05 were deemed to be enriched in DEGs.

**Table 5:** Statistically enriched pathways among the DEGs between MED and Asia II 3.

Pathways	No. of DEGs*	No. of genes**	*p*-value***
Pentose and glucuronate interconversions	13	26	0.001
Galactose metabolism	14	29	0.001
Drug metabolism - other enzymes	17	40	0.002
Oxidative phosphorylation	28	80	0.005
Starch and sucrose metabolism	18	47	0.006
beta-Alanine metabolism	7	15	0.011
Ascorbate and aldarate metabolism	8	18	0.012
Steroid hormone biosynthesis	12	30	0.012
Other types of O-glycan biosynthesis	8	18	0.012
Glyoxylate and dicarboxylate metabolism	5	10	0.014
Porphyrin and chlorophyll metabolism	11	28	0.017
Fructose and mannose metabolism	8	20	0.026
Long-term depression	5	12	0.039
Retinol metabolism	15	45	0.042
Peroxisome	16	49	0.046

* The number of differentially expressed genes (DEGs) that belong to a KEGG pathway.

** The total number of orthologous genes that belong to a KEGG pathway.

*** The significantly enriched pathways were identified using hypergeometric test and pathways with the p≤0.05 were deemed to be enriched in DEGs.

### Differently Regulated Pathways between the Two Invasive Species

**Figure 6:**
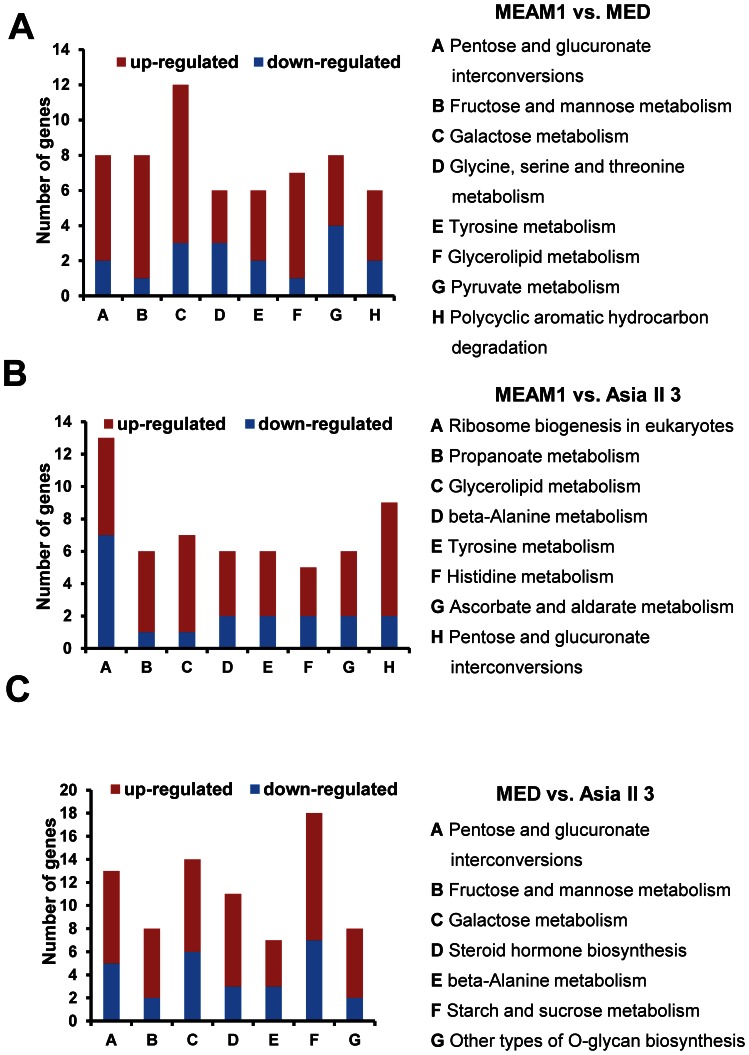
Analysis of DEGs in the enriched carbohydrate, amino acid, glycerolipid and steroid related metabolic pathways among MEAM1, MED, Asia II 3. (A) The number of up-regulated and down-regulated genes in these enriched pathways between MEAM1 and MED. (B) The number of up- and down-regulated genes in these enriched pathways between MEAM1 and Asia II 3. (C) The number of up- and down-regulated genes in these enriched pathways between MED and Asia II 3.

When comparing MEAM1 and MED, the DEGs were significantly enriched in three carbohydrate and two amino acid metabolic pathways (‘Pentose and glucuronate interconversions’, ‘Fructose and mannose metabolism’, ‘Galactose metabolism’, ‘Glycine, serine and theronine metabolism’, and ‘Tyrosine metabolism’) (Table 3). The DEGs involved in these pathways were examined and the results showed that more genes were expressed at a higher level in MEAM1 than in MED, which may indicate the MEAM1 species has higher activities in carbohydrate and amino acid metabolism than MED (Figure 6A). Between the DEGs of the two invasive species, the most enriched pathway was ‘lysosome’ pathway. Interestingly, 25 out of the 28 DEGs involved in this pathway were up-regulated in MEAM1 compared to MED (Table S6). In eukaryotic cells, both endogenous and exogenous macromolecules can be delivered to lysosome for degeneration by more than 50 acid-dependent hydrolases contained within its lumen [53]. The data above implied that the MEAM1 species may have higher lysosome activity than the MED species.

### Differentially Regulated Pathways between Invasive and Indigenous Whiteflies

Regarding MEAM1 vs. Asia II 3, DEGs were significantly enriched in the pathways of carbohydrate and amino acid metabolisms, such as ‘Pentose and glucuronate interconversions’, ‘beta-Alanine metabolism’, ‘Tyrosine metabolism’, ‘Histidine metabolism’ and ‘Glycerolipid metabolism’ (Table 4). For MED vs. Asia II 3, pathways involved in carbohydrate and amino acid metabolisms were significantly enriched with DEGs as well, such as ‘Pentose and glucuronate interconversions’, ‘Fructose and mannose metabolism’, ‘Galactose metabolism’, ‘Starch and sucrose metabolism’, ‘Other types of O-glycan biosynthesis’ and ‘beta-Alanine metabolism’ (Table 5). Among those pathways, the majority of genes were expressed at a higher level in MEAM1 and MED compared to Asia II 3 (Figure 6B, 6C). Carbohydrates, amino acids and glycerolipids are not only the building blocks of insect body, but also essential for various behavioral and physiological processes [54]–[56]. Thus, our data analysis revealed that, on cotton plants, carbohydrate, amino acid and glycerolipid metabolisms might be more active in MEAM1 and MED than in Asia II 3. The diverged expression of metabolism genes in MEAM1, MED and Asia II 3 may contribute to their differences in biological characteristics and adaptability to host plants (Figure 4).

### Up-regulation of Drug Metabolism and Anti-stress Related Genes in MEAM1 and MED

Between MEAM1 and Asia II 3, the DEGs were significantly enriched in the ‘Drug metabolism - other enzymes’ pathway (*p = *0.035), and 9 out of the 11 DEGs were expressed at a higher level in MEAM1 compared to Asia II 3 (Table 6). Among these DEGs, two P450 genes and two UDP-glucuronosyltransferase (UGT) genes were expressed at a higher level in MEAM1 than in Asia II 3 with log_2_ (MEAM1/Asia II 3) >4.2. As for MED vs. Asia II 3, the DEGs were also significantly enriched in the ‘Drug metabolism - other enzymes’ pathway (*p = *0.002), and 12 out of 17 genes were up-regulated in MED compared to Asia II 3. Interestingly, among these DEGs, three P450 genes and three UGT genes were up-regulated in MED compared to Asia II 3 (Table 7). During the interaction between insects and plants, insects have evolved mechanisms to protect themself from plant toxic compounds by a variety of enzymatic detoxification systems. P450s occupy an important position in detoxification processes due to their extraordinary versatility, multiplicity and diversity of substrate recognition sites [57], [58]. In addition, P450s also play a significant role in insecticide resistance [59], [60]. UGTs belong to another family of enzymes involved in insect detoxification of plant allelochemicals and insecticides through glycosylation, which converts lipophilic aglycones into more hydrophilic glycosides, facilitating the excretion or sequestration for further utilization [61], [62]. Both MEAM1 and MED have a wide range of host plant, and MED is also known for its anti-insecticide ability. As P450s and UGTs have those important functions, their high expression in MEAM1 and MED may contribute to the wide host plant range and anti-insecticide ability of both MEAM1 and MED whiteflies [31], [32] and lead to the two species’ invasion and displacement of the indigenous Asia II 3 species.

**Table 6:** DEGs in ‘Drug metabolism - other enzymes’ pathway in MEAM1 vs. Asia II 3.

Gene ID	Homologous function*	Species	Accession no.	FC**
BT_B_ZJU_Singletons26640	UDP-glucuronosyltransferase 1–8 precursor, putative	*Pediculus humanus corporis*	XP_002427365.1	−1.47
BT_Q_ZJU_Singletons12425	Cytochrome P450 6B21	*Papilio glaucus*	AF280622_1	−1.46
BT_B_ZJU_Singletons74969	UDP glucuronosyltransferase 2B11	*Gallus gallus*	XP_420613.2	2.96
BT_B_ZJU_Singletons104121	GMP synthase	*Tribolium castaneum*	XP_001812886.1	3.25
BT_B_ZJU_Singletons103244	Inosine-5'-monophosphate dehydrogenase	*Harpegnathos saltator*	EFN84850.1	3.32
BT_Q_ZJU_Singletons166670	Glucosyl/glucuronosyl transferases	*Tribolium castaneum*	XP_967606.2	3.32
BT_B_ZJU_Singletons101287	Dihydropyrimidine dehydrogenase	*Camponotus floridanus*	EFN63987.1	3.62
BT_B_ZJU_Singletons81386	Cytochrome P450 CYP6CX1v1	*Bemisia tabaci*	ACT68012.1	4.21
BT_B_ZJU_Singletons21983	Feruloyl esterasee protein, Est4	*Reticulitermes flavipes*	ACT53739.1	4.95
BT_Q_ZJU_Singletons121366	UDP-glucosyltransferase	*Bombyx mori*	NP_001182390.1	6.39
BT_B_ZJU_Singletons103451	Cytochrome P450 CYP6CX1v1	*Bemisia tabaci*	ACT68012.1	6.45

* The function of the homologous gene.

** FC, fold change (log_2_ MEAM1/Asia II 3) of gene expression.

**Table 7:** DEGs in ‘Drug metabolism - other enzymes pathway’ in MED vs. Asia II 3.

Gene ID	Homologous function*	Species	Accession no.	FC**
BT_Q_ZJU_Singletons12425	Cytochrome P450 6B21	*Papilio glaucus*	AF280622_1	−6.26
BT_B_ZJU_Singletons1669	Spermatogonial stem-cell renewal factor	*Branchiostoma floridae*	XP_002594939.1	−2.71
BT_Q_ZJU_Singletons29450	UDP-glucuronosyltransferase 2A3 isoform 2	*Pan troglodytes*	XP_526602.2	−1.87
BT_Q_ZJU_Singletons152808	Dihydropyrimidinase, putative	*Pediculus humanus corporis*	XP_002431143.1	−1.82
BT_B_ZJU_Singletons26640	UDP-glucuronosyltransferase 1–8 precursor, putative	*Pediculus humanus corporis*	XP_002427365.1	−1.53
BT_Q_ZJU_Singletons34238	Antennal-enriched UDP-glycosyltransferase	*Tribolium castaneum*	XP_969004.1	1.83
BT_Q_ZJU_Singletons6014	Cytochrome P450 CYP6CX1v2	*Bemisia tabaci*	ACT78507.2	2.33
BT_B_ZJU_Singletons104121	GMP synthase, putativehypothetical protein	*Pediculus humanus corporis*	EFA09313.1	2.87
BT_B_ZJU_Singletons74969	UDP-glucuronosyltransferase 2B11 protein	*Gallus gallus*	XP_420613.2	3.23
BT_ZHJ1_ZJU_Unigene39602	UDP-glucuronosyltransferase 2B7 precursor	*Canis familiaris*	XP_852203.1	3.65
BT_Q_ZJU_Singletons166670	Glucosyl/glucuronosyl transferases	*Tribolium castaneum*	XP_967606.2	3.68
BT_B_ZJU_Singletons101287	Dihydropyrimidine dehydrogenase [NADP+]	*Camponotus floridanus*	EFN63987.1	4.01
BT_B_ZJU_Singletons81386	Cytochrome P450 CYP6CX1v1	*Bemisia tabaci*	ACT68012.1	4.01
BT_B_ZJU_Singletons21983	Feruloyl esterase-like protein Est4	*Reticulitermes flavipes*	ACT53739.1	4.40
BT_ZHJ1_ZJU_Unigene25036	Uridine cytidine kinase	*Nasonia vitripennis*	XP_001605136.1	4.55
BT_B_ZJU_Singletons103451	Cytochrome P450 CYP6CX1v1	*Bemisia tabaci*	ACT68012.1	5.07
BT_Q_ZJU_Singletons121366	UDP-glucosyltransferase protein 3	*Bombyx mori*	NP_001161187.1	6.89

* The function of the homologous gene.

** FC, fold change (log_2_ MED/Asia II 3) of gene expression.

In the ‘Peroxisome’ pathway, a CuZn SOD gene and a catalase gene were found expressed at a higher level in MEAM1 and MED than in Asia II 3 (Table S6). SOD is an important member of antioxidant enzymes capable of detoxifying reactive oxygen species such as superoxide anion (O_2_^−^) and hydrogen peroxide (H_2_O_2_), which were produced under various stress conditions [63]. A series of studies have reported that CuZnSOD involved in defense response against many biological stimulators such as heavy metals [64], heat shock [65], infection and pesticide resistance [66], [67]. Catalase is another antioxidant enzyme responsible for the degradation of H_2_O_2_ generated under stress conditions [68]–[70]. Compared to Asia II 3, MEAM1 is known for its higher adaptive ability to a number of host plants [21], [71], and MED has its own advantage in insecticide resistance [32]. The results above are particularly interesting and suggest that the higher adaptive capacity observed in MEAM1 species or the stronger anti-insecticide ability of MED might be related to the over-expression of some important genes of detoxification or anti-stress.

### Other Differentially Regulated Pathways between the Invasive and Indigenous Species

Besides the differently regulated pathways between the invasive and indigenous whiteflies noted above, our analysis also showed that ‘Ribosome biogenesis in eukaryotes’ and ‘Ascorbate and aldarate metabolism’ were enriched with DEGs in MEAM1 vs. Asia II 3. Ribosome biogenesis underlies the cell's capacity to grow as ribosomes synthesize prodigious numbers of proteins, which is required for cell growth and proliferation [72]. The data showed that 11 out of the 16 genes contained in ‘Ribosome biogenesis’ were up-regulated in MEAM1 compared to Asia II 3 indicating the higher ability of MEAM1 in this pathway (Table S6). In MED vs. Asia II 3, ‘Oxidative phosphorylation’ pathway was significantly regulated. From ‘Oxidative phosphorylation’ pathway, organisms not only acquire the energy that is essential for cellular activities, but also provide precursors for the biosynthesis of amino acids and NADH that is used in numerous biochemical reactions [73]. The different regulation of oxidative phosphorylation may be important for the differentiation of MED and Asia II 3 and warrants further investigations.

In summary, we present, for the first time, an extensive analysis of transcriptional differences of two invasive whitefly species MEAM1, MED and an indigenous whitefly Asia II 3. Our data shows that among the orthologous genes shared by the three species, more DEGs were identified between MED and Asia II 3 than between MED and MEAM1, which corresponds to the results of previous phylogenetic analyses. KEGG enrichment analysis showed that MEAM1, MED and Asia II 3 differ from each other in several pathways related to carbohydrate, amino acid and glycerolipid metabolisms. In addition, most of the drug metabolism and detoxification related genes such as P450s, UGTs, CuZnSOD and catalase were expressed at a higher level in MEAM1 and MED than in Asia II 3. The higher expression of these genes in the invasive MEAM1 and MED species may contribute to their adaptability to a wide range of host plants and detoxification ability. To confirm this observation, the activity of these enzymes should be compared among the invasive and native whitefly species. In addition, we may silence the expression of critical genes and examine whether it will affect the competition between invasive and native whitefly species.

## Supporting Information

Figure S1 **Distribution of distinct tags over different tag abundance categories.** Numbers in the square brackets indicate the range of copy numbers for a specific category of tags.
(PDF)

Table S1 **The expression level of orthologous genes in MEAM1, MED and Asia II 3.**
(XLS)

Table S2 **The DEGs among MEAM1, MED and Asia II 3.** Orthologous genes with the FDR <0.001 and the absolute value of log_2_ Ratio ≥1 were judged to be the different expression genes (DEGs) between species.
(XLS)

Table S3 **Primers for qPCR.** The primers of the 30 selected genes used for validation of expression level and the reference gene (β-actin) were listed.
(XLS)

Table S4 **Data of qPCR validation.** Thirty genes were selected for validation of expression level using qPCR. Log_2_ Ratio_DGE: fold change (log_2_ Ratio) of gene expression in DGE analysis; Log_2_ Ratio_qPCR: fold change (log_2_ Ratio) of gene expression in qPCR; Concordant up: genes were up-regulated both in DGE and qPCR analyses; Concordant down: genes were down-regulated both in DGE and qPCR analyses; Up DGE, down qPCR: genes up-regulated in DGE analysis were down-regulated in qPCR assay; Down DGE, up qPCR: genes down-regulated in DGE analysis were up-regulated in qPCR assay.
(XLS)

Table S5 **Enriched GO terms in ‘Biological process’ category.** No. of DEGs: the number of differentially expressed genes (DEGs) that belong to a KEGG pathway. No. of genes: The total number of orthologous genes that belong to a KEGG pathway.
(XLS)

Table S6 **KEGG annotation of DEGs among the three species.** The KEGG annotation of differentially expressed genes in 1) MEAM1 vs. MED, 2) MEAM1 vs. Asia II 3, and 3) MED vs. Asia II 3 were listed.
(XLS)
