# Supplementary figures and images for: Analysis of the Transcriptional Differences between Indigenous and Invasive Whiteflies Reveals Possible Mechanisms of Whitefly Invasion
Source: PLoS One. 2013 May 8;8(5):e62176. doi: 10.1371/journal.pone.0062176 (PMC3648516; doi:10.1371/journal.pone.0062176)

# Figure S1

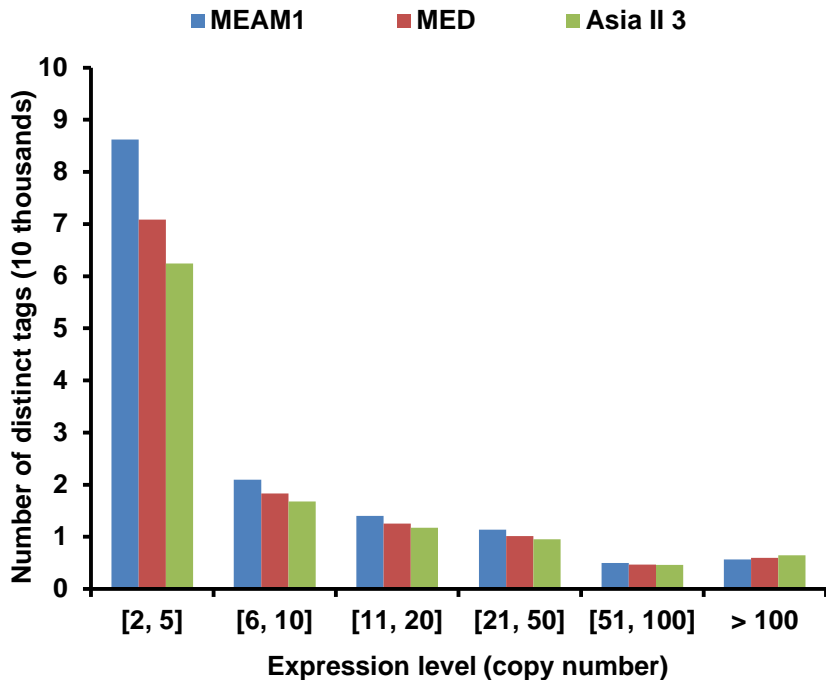

Supplement: Figure S1 — Distribution of distinct tags over different tag abundance categories. Numbers in the square brackets indicate the range of copy numbers for a specific category of tags. (PDF) [file pone.0062176.s001.pdf]
